# Impact of hypoxic air quality on resistance training effect in different normobaric hypoxia environments

**DOI:** 10.1007/s00421-026-06135-7

**Published:** 2026-02-07

**Authors:** Juan Abril, Rafael Timón, Belén Feriche, Cristina Benavente, Paulino Padial, Juan Bonitch-Góngora, Sergio Pérez-Regalado, Filipa Almeida

**Affiliations:** 1 Sports Training and Physical Conditioning Advances Group (GAEDAF), Faculty of Sport Sciences, University of Extremadura, Cáceres, 10003 Spain; 2 Department of Physical Education and Sport, Faculty of Sport Sciences, University of Granada, Granada, Spain; 3 Sport and Health University Research Institute (iMUDS), University of Granada, Granada, Spain

**Keywords:** Hypoxia, Strength, Hypertrophy, Carbonic levels, Simulated hypoxia

## Abstract

**Purpose:**

This study compared the impact of hypoxic air quality breathing in two normobaric hypoxia (NH) environments (a tent with high carbonic levels and relative humidity [NHTent] vs. a room with normal carbonic levels and relative humidity [NHRoom]) on the outcomes of an 8-week resistance training (R_T_) program.

**Methods:**

Twenty-four trained men (age: 22 ± 3 years; weight: 76.32 ± 11.01 kg; height: 176.79 ± 7.48 cm) were assigned to either the NHTent or the NHRoom group being exposed to the same FiO_2_ (15.9%) to analyze the impact of two different procedures to generate intermittent NH on structural, physiological, functional, and perceptual responses after a R_T_ program. CO_2_ and relative humidity levels were measured before and after each training session. Physiological variables (heart rate [HR] and SpO_2_) were monitored and used for comparison between the first and last training sessions. Functional (bench press and squat 1RM) and structural (vastus lateralis thickness) responses were measured before and after the program.

**Results:**

CO_2_ and relative humidity levels were higher in the NHTent (*p* < 0.001). Compared to NHRoom, NHTent group displayed higher HR (*p* = 0.002), lower SpO_2_ (*p* = 0.014), greater increases in 1RM (*p* = 0.011) and lower increases in vastus lateralis thickness (*p* = 0.06).

**Conclusion:**

These findings suggest that the CO_2_ and relative humidity levels on the hypoxic air breathed during training alter the physiological response to the R_T_. The harsher environment seems to enhance neuromuscular adaptations but limit hypertrophic responses. Results highlight the need to control environmental factors carefully when simulating NH conditions for training purposes.

## Introduction

Skeletal muscle adapts structurally and functionally in response to different stimuli, such as the combination of resistance training (R_T_) and systemic hypoxic conditions[1]. Exposure to systemic hypoxia can be achieved by reducing the barometric pressure which results in hypobaric hypoxia (HH) [2] or by reducing the inspired fraction of oxygen (FiO_2_) without changes in the barometric pressure, which results in normobaric hypoxia (NH) [3]. In both conditions, the severity of hypoxia is determined by the inspired oxygen partial pressure (PiO₂), which depends on both barometric pressure and FiO₂ [4]. According to Koehle et al. [5], hypoxia is common classified in four levels: low (< 1500 m; FiO_2_ > 18%), moderate (1500–3000 m; FiO_2_ = 18–15%), severe (3000–5000 m; FiO_2_ = 15–11%) and highly severe (> 5000 m; FiO_2_ < 11%). Nevertheless, other factors may impact the severity of the internal hypoxia, such as environmental carbonic (CO_2_) levels resulting from the athletes’ exhalation and energy expenditure throughout the exercise and, to a lesser extent, the increase in the environmental temperature and relative humidity [6].Therefore, the dimensions of the facilities and the devices used to simulate hypoxia, as well as the air exchange rate associated with these devices, determine the CO_2_ and relative humidity levels on the air breathed inside the tent or room. These factors may constitute a bias in the current literature because these aspects have not been studied yet, nor have their potential effect on certain physiological parameters been considered. Among them, alveolar pressure of oxygen (PAO_2_), which can be affected by the respiratory exchange ratio (R) or the alveolar pressure of carbon dioxide (PACO_2_) (PAO_2_ = FiO_2_ [PB-47] − 1/R [PACO_2_]). Where R usually equals 0.8 and the 47 corresponds to the water vapor pressure at normal body temperature (37 °C) [7]. Most studies that combine R_T_ with NH conditions [8] use tents or rooms and do not control or indicate the aspects previously described. Only a few studies on repeated-sprint training in hypoxia (RSH) have controlled for the environmental factors described[9].

A hypoxic environment may induce beneficial responses in hypertrophic adaptations [10]. Metabolic stress, cellular swelling, and an increase in the production of myokines and reactive oxygen species in response to metabolite accumulation have been considered important regulators of muscle adaptive response [11]. When combined with resistance exercise, hypoxia may further amplify the accumulation of metabolites such as lactate, calcium, and inorganic phosphate, which in turn can stimulate hypertrophic pathways through elevated anabolic hormone secretion (e.g., growth hormone), enhanced cell swelling, and modulation of local myokines (e.g., IL-6, IL-10, myostatin). These metabolic and molecular responses may be more pronounced under hypoxic conditions, potentially resulting in greater hypertrophic outcomes compared with equivalent training performed in normoxia [12–15]. Thus, it is conceivable that R_T_ under moderate hypoxia may improve muscle growth and strength compared with the same training under normoxia conditions. However, the last available meta-analysis on R_T_ in hypoxia [16], alongside others [17], did not reveal remarkable differences in muscle growth and strength development between NH and normoxia, probably because of the great disparity in the protocols used. Loads between 60 and 80% 1-repetition maximum (1RM), inter-set rest intervals of ≤ 60 s, and moderate hypoxia seem to enhance muscle growth, whereas loads between 60 and 80% 1RM, inter-set rest intervals ≥ 120 s, and moderate or severe hypoxia seem to enhance strength gains [16]. However, another factor that can also be related to the lack of consensus is the manner in which NH conditions are generated (i.e., using NH systems / devices which originate additional adverse environmental conditions to the inspired FiO_2_)[16]. The combination of lower oxygen availability and increased environmental stress factors may affect performance[11]. Consequently, designing a suitable training methodology (loads and inter-set rest intervals) and controlling the environmental conditions (temperature, relative humidity and CO_2_ levels) are presented as two determining groups of factors in achieving the best results after a R_T_ under hypoxia condition. Therefore, this study aimed to analyze the impact of two different procedures to generate intermittent systemic NH (at the same FiO_2_ but with different CO_2_ and relative humidity levels) on muscle growth, strength, heart rate (HR), arterial (SpO_2_) and muscle oxygen saturation (SmO_2_) and rating of perceived exertion (RPE) responses after an 8-week R_T_. We hypothesized that the worse air quality (higher relative humidity and CO_2_) would induce higher metabolic stress, improving muscle mass and strength development.

## Materials and methods

### Experimental approach to the problem

**Fig. 1:**
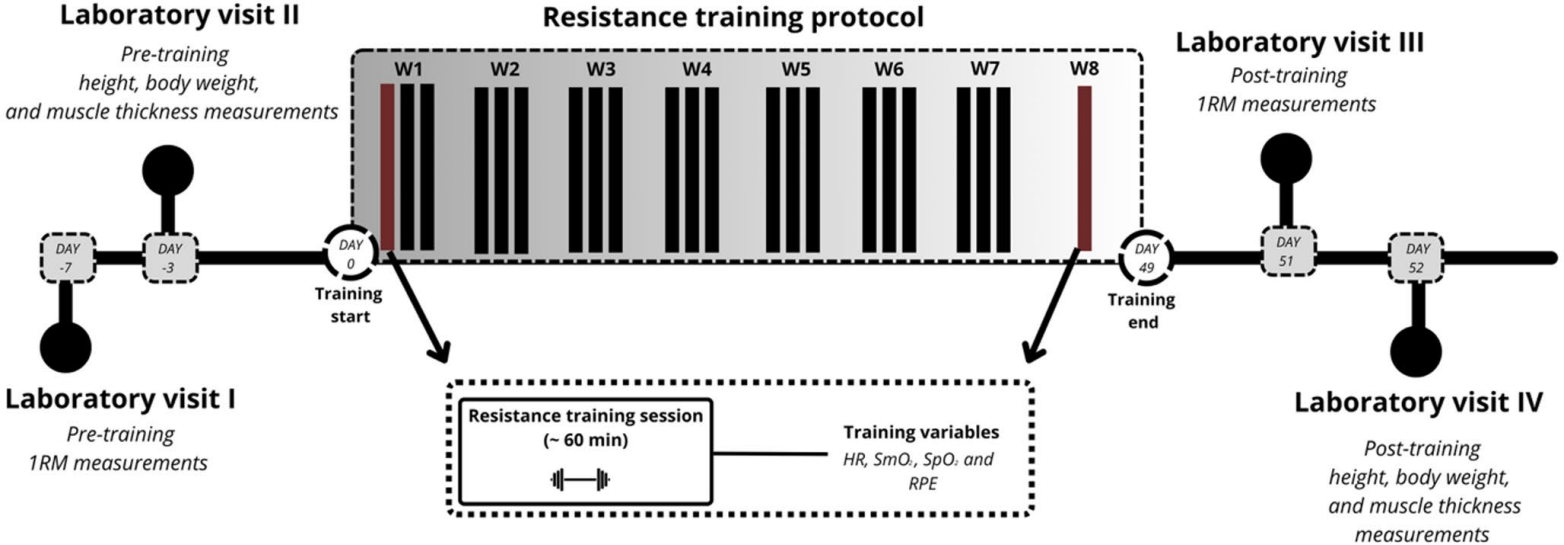
Schematic overview of the training protocol and measurements obtained before and after the intervention in all groups. W: week; HR: heart rate; SmO_2_: muscular oxygen saturation; SpO_2_: arterial oxygen saturation; RPE: rating of perceived exertion; NHTent: group that trained in tent (poor air quality); NHRoom: group that trained in room (good air quality); 1RM: one maximum repetition; FiO_2_: fraction of inspired oxygen

We employed a longitudinal design, with intra- and inter-group measurements, to analyze the impact of two different environmental conditions under the same moderate NH (high vs. low CO_2_ and relative humidity levels, i.e., tent vs. room) on strength, muscle mass, perceptual and physiological responses after a R_T_ period (Fig. 1). Thus, two homogenous groups trained under similar intermittent NH conditions (FiO_2_ = 15.9%, ~ 760 mmHg), one group in a tent (NHTent, *n* = 13), and the other in a room (NHRoom, *n* = 11) for 8 weeks. All participants lived under normoxic conditions. Height (Seca 202, Seca Ltd, Hamburg, Germany), body mass (Tanita TBC-300, Tokyo, Japan), vastus lateralis muscle thickness, and 1RM in squat and bench press were measured 72 h before and after the study. HR, SpO_2_ and SmO_2_ and RPE were measured in the first and last training sessions. CO_2_ and relative humidity levels were measured before and after each training session.

Participants were instructed to maintain their life and eating habits during the study. After all training sessions, they all received a protein shake (Life Pro, 109.9 kcal, 0.4 fats, 1 g carbohydrates and 25.2 g protein per serving) to ensure an adequate intake of high-quality proteins. Each participant trained at the same hour of the day, and FiO_2_, barometric pressure and temperature were constant. The relative humidity and CO_2_ levels were measured to control daily variations.

### Participants

Twenty-four healthy young men participated in the study, divided into two homogenous groups. One group trained in a tent (NHTent; *n* = 13, age: 22 ± 2 years; weight: 75.02 ± 8.89 kg; height: 176.53 ± 7.44 cm) and the other group trained in a room (NHRoom; *n* = 11, age: 23 ± 4 years; weight: 77.85 ± 13.37 kg; height: 177.09 ± 7.86 cm). All participants had experience in R_T_ and had been training at least three times per week during the last two years. They knew the movement’s basic patterns and demonstrated an adequate exercise technique. All participants self-reported no chronic diseases or recent injuries that could compromise performance. They were not smokers, nor did they take any medicines or anabolic agents. They lived at sea level and were not exposed to altitudes or hypoxic environments greater than 1500 m in the last two months before the study.

### Exposure to hypoxia

The participants completed the R_T_ program under intermittent corresponding NH conditions. Participants from the NHTent performed all the training sessions in a NH tent of 9 m^2^, with a total volume of approximately 32 m^3^ (CAT 310, Colorado Altitude Training, USA). A hypoxic generator created the hypoxic environment using a semi-permeable filtration membrane (nitrogen filtration technique; CAT 12, Colorado Altitude Training, USA). The hypoxic system pumped the air with an air flux of 100 L/min to the NH tent to reduce the O_2_ content. FiO_2_ was continuously controlled with a digital recorder (Handi+, Maxtec, Salt Lake City, Utah, USA) to maintain stable hypoxic conditions. Before and after each training session, relative humidity and CO_2_ levels were measured using a digital recorder (Green Eye, TechGrow, The Hague, Netherlands). The NHRoom group completed the R_T_ program in a NH room of 60 m^2^ and a total volume of 240 m^3^ (Fusiotech SARL, Ventte, France). The hypoxic system was generated by O_2_ extraction with 3 control points to monitor O_2_ levels using zirconium oxide. CO_2_ levels were measured by a non-dispersive infrared sensor and an electrolytic cell. The room hypoxic device allows a mean ventilation of 3400 L/min. The room has an integrated control system that displays FiO_2_, relative humidity and CO_2_ in real time. The temperature in the room was maintained at 22 ºC using an air-conditioning device, while in the tent was 23.0 ± 1.04 °C (Green Eye, TechGrow, The Hague, Netherlands). The barometric pressure was not manipulated during the study and was ~ 760 mmHg for both groups. The FiO_2_ was the same for the tent and room (FiO_2_ = 15.9%).

### Resistance training program

**Fig. 2:**
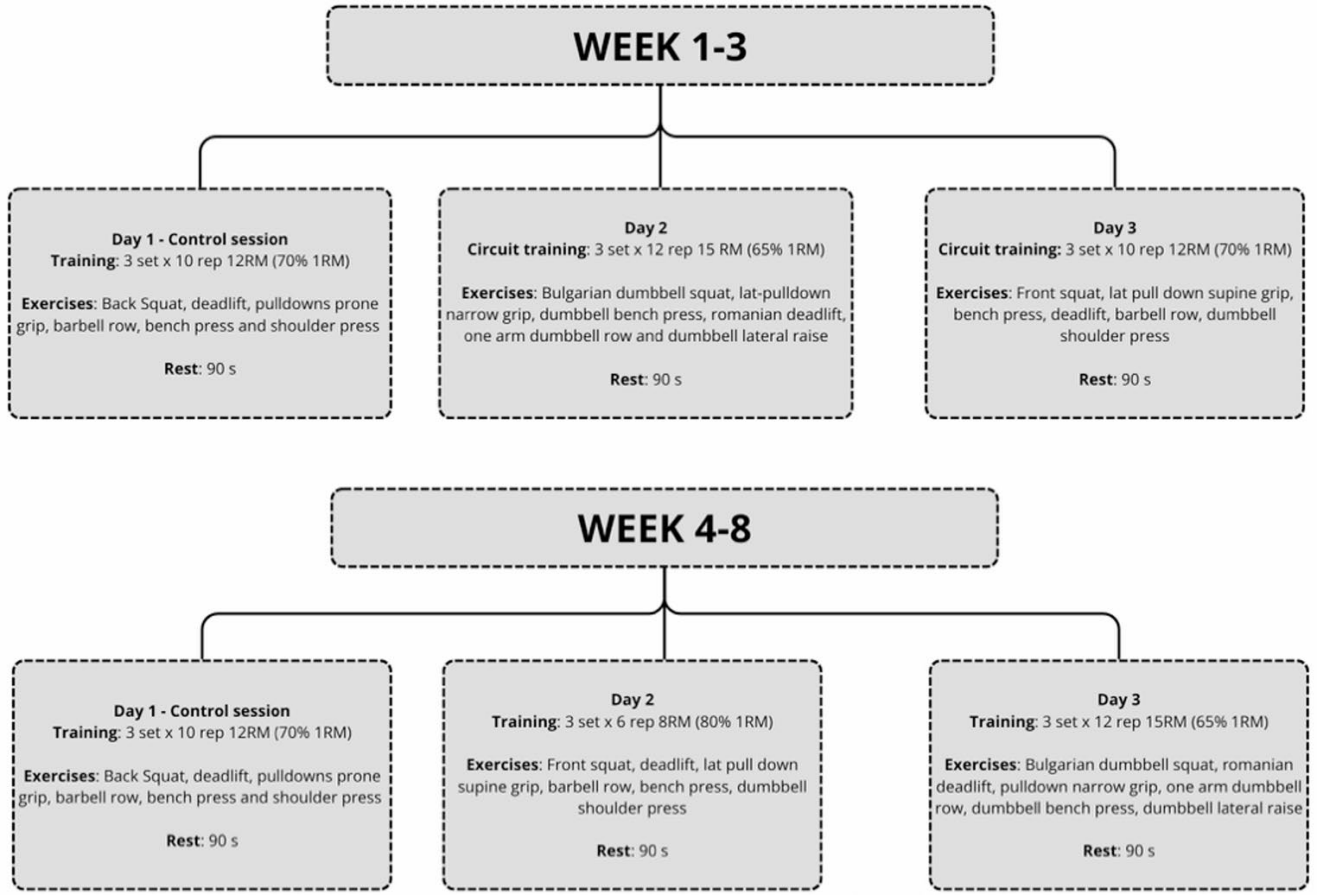
Training regime for the 8 weeks of the resistance training program

Participants completed an 8-week R_T_ program with 3 sessions per week, comprising a total of 22 sessions. They performed a 15-min standardized warm-up in NH conditions before each session (5 min of cardiovascular activation in a cycle-ergometer, 5 min of joint mobility and dynamic stretching and 5 min of specific warm-up). The R_T_ session encompassed the execution of 6 exercises, with three sets of 8 to 12 repetitions per exercise (65–80% 1RM), leaving 1 to 2 repetitions in reserve per exercise, and 90 s of rest between sets and exercises (Fig. 2). Participants rested 48 h between them and had an extra day of rest at the end of the week. The training load was individually adjusted weekly to maintain the same mechanical stimuli throughout the training period.

### Training control

HR, SmO_2_, SpO_2_ and RPE were monitored during the first weekly session of the training program (control session). Data from the first (S1) and last (S22) training sessions were compared.

The HR was continuously recorded beat-by-beat using an HR belt (Polar H10; Polar Electro Oy, Kempele, Finland) connected to a watch (Polar s610i; Polar Electro Oy, Kempele, Finland). The mean HR from the session, excluding the warm-up and recovery, was calculated.

SmO_2_ was measured during the first exercise of the session (back squat) using near-infrared spectroscopy (NIRS; Moxy, Fortiori Desing, Minneapolis, Minnesota, EEUU) [18]. During all testing, the system was connected to a personal computer via software (Seego, Realtrack Systems, Almería, Spain) that provided a graphic display of the data. The sensor was placed on the vastus lateralis of the dominant leg, halfway between the greater trochanter and lateral epicondyle of the femur before warm-up. The skin was marked with a semi-permanent pen to reproduce the exact location in subsequent tests. To prevent issues with movement during exercise, the device was fixed to the leg with tape and wrapped with a dark elastic bandage, following Scott et al.[19] recommendations. Maximal (SmO_2_max) and minimal (SmO_2_min) values were recorded for each set of the exercise. The relative saturation values were then calculated with respect to the initial value obtained at rest before any procedure (SmO_2_baseline) as follows:

$$\Delta {\mathrm{SmO}}_{{\mathrm{2}}} {\text{max }} = {\text{ SmO}}_{{\mathrm{2}}} {\text{max }} - {\text{ SmO}}_{{\mathrm{2}}} {\text{baseline }}\left( {{\mathrm{resaturation}}} \right)$$

$$\Delta {\mathrm{SmO}}_{{\mathrm{2}}} {\text{min }} = {\text{ SmO}}_{{\mathrm{2}}} {\text{min }} - {\text{ SmO}}_{{\mathrm{2}}} {\text{baseline }}\left( {{\mathrm{desaturation}}} \right)$$

SpO_2_ was measured in duplicate after 5 min of hypoxia exposure and immediately after finishing the session using a pulse oximeter fingertip sensor (Wristox 3100; Nonin, Plymouth, MN, USA). The mean value of each measure was calculated and used in the analysis.

The RPE was assessed 15 min after the completion of the R_T_ session using the Category Ratio-10 scale [20].

### One repetition maximum

Before and after the R_T_ program, the 1RM of the back squat and bench press were calculated for each participant according to the National Strength and Conditioning Association guidelines[21]. Prior to testing, participants performed a warm-up consisting of light cardiovascular exercise lasting 5–10 min, followed by a set of five repetitions at ~ 50% of their estimated 1RM and, afterwards, one or two sets more of two or three repetitions at a load corresponding to ~ 60–80% of the estimated 1RM for the exercise. Three sets of 3–6 repetitions at increasing loads were completed before performing one set of two or three repetitions to failure. The 2–3 RM load was used for 1RM estimation from the validated Brzycki equation[22]. The participants rested for 5 min between each successive attempt. The 1RM determinations were made within 5 attempts.

### Vastus lateralis thickness

Muscle thickness of the vastus lateralis on the dominant leg was measured by one expert using ultrasound equipment (GELOGICQ-E portable model; GE Healthcare, Little Chalfont, UK) before and after the R_T_ program. In accordance with another study [23], the quadriceps were chosen for muscular thickness assessment as they are prime movers in the squat exercise, which was used to quantify changes in strength from the intervention. The maximum thickness of the vastus lateralis was obtained at 50% of the distance from the superior and middle tip of the patella to the anterior superior iliac spine. The lateral location of the vastus lateralis measurement was taken at 10% of the thigh circumference in the lateral direction. With the participant laid supine, the ultrasound probe (12 L linear probe at 10 MHz frequency, gain 80 dB, depth 8 cm) was orientated perpendicular to the muscle fascicles and the skin, with sufficient ultrasound gel to reduce muscle compression. The depth of the image was adjusted until the femur and muscle boundaries were visible on the screen. Three images of vastus lateralis were taken and saved for subsequent analysis. The thickness of the vastus lateralis was defined as the distance from the subcutaneous adipose tissue-muscle interface to either the aponeurosis or the muscle-bone interface. The same expert carried out all ultrasound measurements (CV < 1.8%) and was blinded to which condition each participant was assigned.

### Statistical analysis

Data are presented as mean ± standard deviation (SD). Data normality assumptions were tested using the Shapiro–Wilk test (*p* < 0.05).

Independent-sample t-tests were used to compare the post-session environmental conditions in both chambers.

A two-factor mixed ANOVA was used to assess the effects of time (within-participant factor: first session vs. last session) and environmental conditions (between-participant factor: NHTent vs. NHRoom) on HR, RPE, SmO_2_ and SpO_2_. Partial eta squared for main effects was calculated from the ANOVA (η_p_^2^) and was interpreted as ≥ 0.01 (small), ≥ 0.06 (medium) and ≥ 0.14 (large)[24]. Significant main effects and interactions were subsequently analyzed with the Bonferroni post hoc test.

When comparing the effect of training in NHTent vs. NHRoom in 1RM and muscle thickness, the comparisons were adjusted to the baseline by comparing the post – pre values of the NHTent to the post – pre values of NHRoom (ΔNHTent - ΔNHRoom) using independent-sample t-tests. Additionally, paired-sample t-tests were used to evaluate the intra-group training effect.

Complementary to the previous tests, Cohen’s d effect sizes (ES) were calculated by dividing the mean difference (NHTent - NHRoom or post – pre) by the pooled standard deviations. ES were interpreted using the following classification, as per Hopkins et al.[25]: < 0.2 (trivial), 0.21–0.5 (small), 0.5–0.8 (moderate), 0.8–1.3 (large), and > 1.30 (very large).

SPSS software version 28.0 (IBM Corp., IBM SPSS Statistics for Windows, Armonk, NY, USA) was used for all analyses. The significance level was set at *p* < 0.10.

## Results

A total of 24 participants completed the intervention and their results were included in the analysis. There were no research-related adverse effects or injuries throughout the study. Both groups had similar exposure to NH in each R_T_ session (80 min and FiO_2_ = 15.9%). There were no differences between groups in any pre-test measurement.

### Environmental conditions

**Fig. 3:**
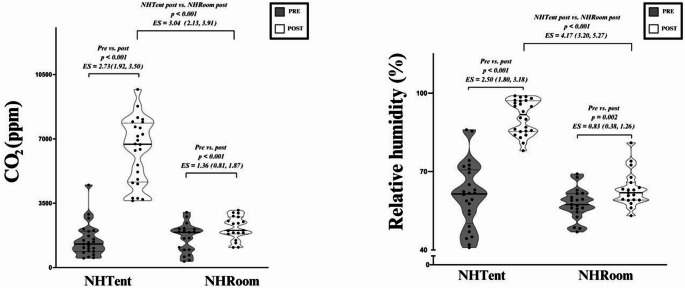
Changes in CO_2_ levels and in relative humidity levels before and after the R_T_ session in NHTent and NHRoom. Intra-group comparison between pre- and post-training session values and inter-group comparison between post values in NHTent vs. NHRoom. NHTent: normobaric hypoxia in the tent; NHRoom: normobaric hypoxia in the room; RH: relative humidity; CO_2_: carbon dioxide; Pre: beginning of the training sessions; Post: end of the training sessions. The black lines represent the median and interquartile range (lower and upper dash lines). ES: effect size was calculated by dividing the mean difference (NHTent – NHRoom or post – pre) by the pooled standard deviations (90% CI)

Results showed significant differences between groups in the post-session CO_2_ levels (*p* < 0.001, ES = 3.04 [2.13, 3.91]), being higher in the NHTent group compared with NHRoom (6374 ± 1766 ppm vs. 2158 ± 629 ppm) (Fig. 3). Results also showed significant differences between groups in the post-session relative humidity levels (*p* < 0.001, ES = 4.17 [3.02, 5.27]), being higher in the NHTent group compared with NHRoom (90.9 ± 6.57% vs. 63.3 ± 6.69%) (Fig. 3).

### One repetition maximum

**Fig. 4:**
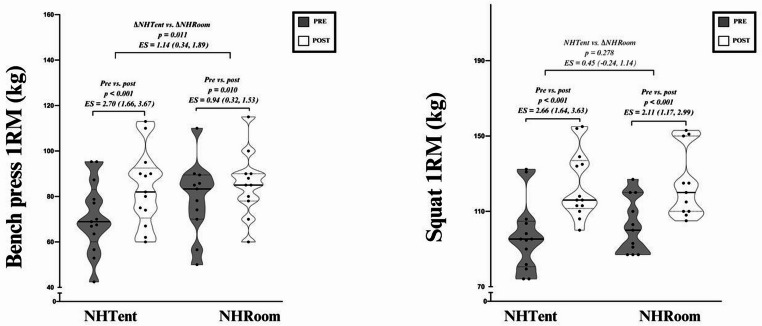
Changes in 1RM bench press and squat values before and after the R_T_ program in NHTent and NHRoom. Intra-group comparison between pre and post R_T_ program values and inter-group comparison adjusted to the baseline between NHTent vs. NHRoom (NHTent – NHRoom). NHTent: normobaric hypoxia simulated in a tent; NHRoom: normobaric hypoxia simulated in a room; 1RM: 1 repetition maximum; Pre: before R_T_ program; Post: after R_T_ program. The black lines represent the median and inter-quartile range (lower and upper dash lines). Dots represent single-subject data. ES: effect size was calculated by dividing the mean difference (NHTent – NHRoom or post – pre) by the pooled standard deviations (90% CI)

Compared with the pretest, NHTent showed significantly higher increases after the R_T_ for the bench press 1RM (12.70 ± 1.30 kg, *p* < 0.001, ES = 2.70 [1.66, 3.67]) than the NHRoom (6.24 ± 1.98 kg, *p* = 0.010, ES = 0.94 [0.32, 1.53]) (mean difference = 6.42 kg [2.46, 10.4 kg], *p* = 0.011, ES = 1.14 [0.35, 1.90]) (Fig. 4).

Concerning the squat 1RM, both groups also improved their results after the R_T_ period (NHTent: 27.2 kg ± 2.83 kg, *p* < 0.001, ES = 2.66 [1.64, 3.63]; NHRoom: 22.4 kg ± 3.21 kg, *p* < 0.001, ES = 2.11 [1.17, 2.99]). Although no significant differences between conditions were found (*p* = 0.278, ES = 0.456 [– 0.24, 1.14]), the adjusted between-group difference favored the NHTent (4.74 kg), with confidence intervals (CI) estimates ranging from a 2.58 kg benefit to the NHRoom to a 12.07 kg benefit to the NHTent (Fig. 4).

### Vastus lateralis thickness

**Fig. 5:**
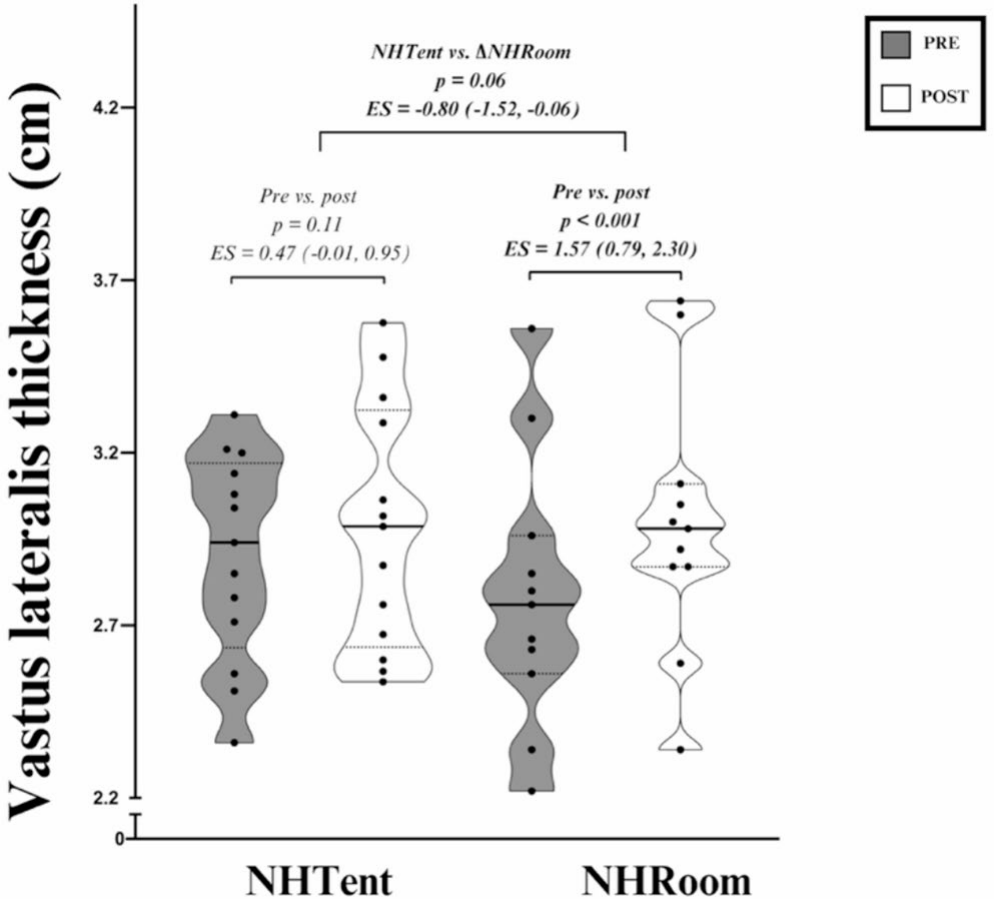
Changes in vastus lateralis thickness values before and after the R_T_ program in NHTent and NHRoom. Intra-group comparison between pre and post R_T_ program values and inter-group comparison adjusted to the baseline between NHTent vs. NHRoom (NHTent – NHRoom). NHTent: normobaric hypoxia simulated in a tent; NHRoom: normobaric hypoxia simulated in a room; Pre: before R_T_ program; Post: after R_T_ program. The black lines represent the median and interquartile range (lower and upper dash lines). Dots represent single-subject data. ES: effect size was calculated by dividing the mean difference (NHTent – NHRoom or post – pre) by the pooled standard deviations (90% CI)

Compared with the pretest, NHRoom showed higher increases after the R_T_ for the vastus lateralis thickness (0.21 ± 0.18 cm, *p* < 0.001, ES = 1.57 [0.79, 2.30]) than NHTent (0.08 ± 0.17 cm, *p* = 0.11, ES = 0.47 [-0.01, 0.95]) (mean difference = – 0.12 cm [– 0.24, – 0.01 cm], *p* = 0.06, ES = – 0.80 [– 1.52, – 0.06]) (Fig. 5).

### Heart rate, rating of perceived exertion, muscular oxygen saturation and arterial oxygen saturation

Table 1 shows the mean ± SD of the intra- and inter-group comparisons and main effects results for HR, RPE, SmO_2_ and SpO_2_.

**Table 1:** Heart rate, rating of perceived exertion, muscular oxygen saturation and arterial oxygen saturation intra- and inter-group comparisons and main effects

Variable	Time-point	NHTent (mean ± SD)	NHRoom (mean ± SD)	Condition		Time		Time × condition	
				p	η²_p_	p	η²_p_	p	η²_p_
HR (bpm)	First session	151 ± 14.6 **	134 ± 12.4	**0.014**	0.243	0.918	0.000	0.111	0.111
	Last session	147 ± 12.5	138 ± 16.2						
RPE	First session	7.23 ± 0.8	7.64 ± 1.03	0.697	0.007	**0.097**	0.120	0.271	0.055
	Last session	7.08 ± 0.8	6.91 ± 1.2						
SmO_2_ minimal values (%)	First session	– 50.9 ± 19.4	– 50.3 ± 17.1	0.676	0.009	0.765	0.005	0.528	0.021
	Last session	– 54.6 ± 21.0	– 49.0 ± 11.8						
SmO_2_ maximal values (%)	First session	7.09 ± 7.8	10.4 ± 6.3	0.144	0.109	0.702	0.008	0.289	0.059
	Last session	– 1.18 ± 30.5	14.3 ± 10.9						
SmO_2_ difference (%)	First session	58.0 ± 17.2	60.7 ± 17.9	0.299	0.057	0.863	0.002	0.533	0.021
	Last session	53.4 ± 23.7	63.3 ± 5.4						
SpO_2_ pre-session (%)	First session	94.1 ± 2.2	93.7 ± 1.1	0.341	0.048	0.827	0.003	0.363	0.044
	Last session	94.1 ± 1.8	94.5 ± 1.7						
SpO_2_ post-session (%)	First session	92.8 ± 1.7	94.2 ± 1.0	**0.018**	0.239	0.774	0.004	0.586	0.014
	Last session	93.3 ± 1.4	94.1 ± 1.2						

SpO_2_: arterial oxygen saturation; RPE: rating of perceived exertion; SmO_2_: muscular oxygen saturation; NHTent: normobaric hypoxia simulated in a tent; NHRoom: normobaric hypoxia simulated in a room. *: NHTent vs. NHRoom difference (*p* < 0.10); **: NHTent vs. NHRoom difference (*p* < 0.05); ^#^: Post vs. Pre difference (*p* < 0.10); ^##^: Post vs. Pre difference (*p* < 0.05)

Significant main effects are shown in bold (p < 0.10).

HR revealed a large environmental effect (*p* = 0.014; η²_p_ = 0.24). Pairwise comparisons showed higher HR in NHTent than in NHRoom (mean difference = 13.5 bpm [6.77, 20.2]; ES = 0.97; *p* = 0.002). Furthermore, NHTent showed higher HR than NHRoom in the first session (mean difference = 17.90 bpm [8.29, 27.5]; ES = 1.31; *p* = 0.004).

RPE showed a moderate time effect (*p* = 0.097; η²_p_ = 0.12). Pairwise comparisons revealed lower RPE values after the R_T_ program compared with pre-test (mean difference = – 0.44 [– 0.88, – 0.004]; ES = − 0.33; *p* = 0.097).

SpO_2_ post-session values revealed a large environmental effect (*p* = 0.018; η²*p* = 0.24). Pairwise comparisons revealed lower SpO_2_ in NHTent than in NHRoom (mean difference = – 1.02% [– 1.70, – 0.34]; ES = − 0.75; *p* = 0.014).

## Discussion

The main aim of this study was to analyze the influence of environmental conditions on the quality of the breathed air and its impact on the functional, physiological and perceptual responses, according to the device used to simulate NH during an 8-week R_T_ program in trained young male participants. CO_2_ levels and relative humidity were clearly higher in the NHTent compared with NHRoom. Bench press 1RM showed greater increases in NHTent compared with NHRoom. Squat 1RM results also favored the NHTent although the data did not reach statistical significance. In contrast, the vastus lateralis thickness exhibited a greater increase in the NHRoom. Furthermore, there was a large environment effect either on HR and SpO_2_, displaying greater HR and lower SpO_2_ in NHTent compared with NHRoom group. These findings indicate that the environmental conditions within the NHTent were more challenging. Nonetheless, participants in the NHTent group displayed greater strength gains despite exhibiting lower muscle growth compared with those in the NHRoom.

Regarding the environmental conditions, the increase in CO_2_ and relative humidity levels was significantly higher during the training sessions in the tent compared with those in the room. These results are in accordance with a recent study [26] in which the CO_2_ levels inside a tent were significantly higher than those displayed while wearing a facial mask, showing a mean above 4000 ppm and reaching values higher than 6000 ppm. Similarly, in our study, the mean CO_2_ concentration in the tent reached 6374 ppm, exceeding 9000 ppm in certain sessions, while it only reached an average of 2158 ppm in the room. Exhalation and energy expenditure increase the CO_2_ levels 27], relative humidity , and environmental temperature [28] during training sessions. If the environmental temperature is not controlled, it would probably cause a rise in body temperature, preventing thermoregulation at a neural level that could affect the thermal comfort during the training session [29]. Therefore, in our study, the environmental temperature was at 22º C in the NHRoom and 23.0 ± 1.04° C in the NHTent. If the ventilation of the facility is not adequate, as occurs in the NHTent, the air quality will probably be considerably worsened due to the impossibility of proper air extraction and renewal. Training while re-breathing air previously exhaled may trigger hypercapnic hypoxia and increase respiratory frequency[30]. This is caused by inadequate O_2_, and CO_2_ exchange, increasing the CO_2_ arterial concentration that, combined with the NH condition, may exacerbate the increment of the metabolic acidosis[31]. Additionally, exercising in an enclosed space could increase the partial pressure of CO_2_ , which leads to a higher inspired fraction of CO_2_, negatively affecting physiological responses[32] . Currently, it is recommended that the CO_2_ levels during exercise do not exceed 3000 ppm[33]. Therefore, this recommendation was accomplished only during the training sessions in the NHRoom. Usually, indoor concentrations are 700 ppm above outdoor concentrations (350 ppm), reaching 2800 ppm without causing severe symptoms[34]. CO_2_ levels between 500 and 5000 ppm cause an additional workload on the respiratory system[35], while levels between 2000 and 5000 ppm potentially affect performance and health [45].

Our study showed a functional adaptation to training in both groups, especially in NHTent despite the adverse conditions of humidity and CO_2_ breathed. The NHTent reached greater gains either in the bench press and in the squat 1RM (Fig. 4). We failed to reach significance in the squat exercise, probably because of the high intra-group variability in exercises that lift such heavy loads. In contrast, the vastus lateralis thickness only increased in the NHRoom. The NHTent group did not show improvements, which is in accordance with Kurobe et al.[36], that studied trained participants who underwent a R_T_ program under more severe NH conditions in a similar tent (FiO_2_: 12.7%). The higher carbonic levels displayed in the NHTent may explain the limitation in vastus lateralis gains observed in this group. The impact of exposure to higher CO_2_ levels reduces the alveolar pressure of O_2_, increasing the tissue hypoxia for the same FiO_2_ and potentially affecting the upregulation of the muscle myogenic pathways [37]. Therefore, the challenging conditions experienced during training in the NHTent seem to increase the physiological stress, resembling the environment at high altitudes (4000–5000 m) more closely than at moderate altitudes. At such high altitudes, neural adaptations tend to be prioritized over structural adaptations [38]. This may occur because of the need to preserve skeletal muscle regeneration in these conditions[43]. In this line, Benavente et al.[23] also found that training in NH in a tent favors neural adaptations compared to training under the same FiO_2_ conditions at terrestrial altitude.

Regarding the metabolic responses, the harsher environmental conditions in NHTent have indeed affected the HR and SpO_2_. An environment effect was observed on HR, being higher in NHTent compared to NHRoom and reaching a mean difference of 13.5 bpm. This is consistent with a recent meta-analysis [39], which found that HR was affected by hypoxia conditions after analyzing 61 studies. It showed that both hypoxemia exposure duration and arterial CO_2_ and/or pH have a profound effect on neuronal and cardiovascular adaptation to hypoxemia[39]. These values may be affected by air CO_2_ concentrations, aggravating the severity of hypoxia and therefore impacting the HR response. Similarly, there was a significant environment effect on SpO_2_, being lower in NHTent compared with NHRoom and reaching a mean difference of -1%. This was predictable due to the fact that although the FiO_2_ was the same for both conditions, the environmental conditions in NHTent were harsher compared to NHRoom. This finding aligns with previous research [26], which reported that SpO₂ was influenced by the environmental condition, being lower in the hypoxia tent compared with other NH conditions. Contrary to what was expected, harsher environmental conditions in NHTent did not affect SmO_2_ values. According to Yamaguchi et al. ,[44] hypoxic conditions with lower FiO_2_ aggravate the effects induced by exercise on the muscle deoxygenation response. This may have been the case in our study since the higher levels of CO_2_ seem to induce lower internal hypoxia. Unexpectedly, this did not occur in our study.

Despite the unfavorable environmental conditions in NHTent, we did not detect an effect on the RPE of this group. Thus, training in these conditions of high CO_2_ concentrations and relative humidity did not appear to affect the individual perception of effort significantly. The lack of differences between groups in the RPE may be due to the fact that both groups trained at the same intensity[40] in both conditions. Progressive arterial hypoxemia and the increase in ventilation have been identified as the main indicators to determine the RPE variation under moderate hypoxia conditions [41]. Therefore, although the mean SpO_2_ values displayed at the end of the sessions in NHTent were lower compared to NHRoom (Table 1), it does not seem to be sufficient to generate a change in the perceptive responses of the participants. Additionally, there was a significant time effect on RPE, being lower after the R_T_ program. These results are in line with Brocherie et al.[42] study, in which an attenuation of RPE after hypoxic training was described, suggesting a better tolerance or acclimatization to hypoxia after a single session.

This research has some limitations that should be noted. First, the sample size was relatively small, which may have affected the range of probability distributions for the outcomes. The duration of the study and the specific characteristics of the sample limited the recruitment of a larger sample. However, the results from this population remain particularly relevant. Second, muscle thickness and SmO_2_ were measured only in a small portion of the vastus lateralis, which may hinder the possibility of coming to strong conclusions on these variables.

In conclusion, R_T_ under moderate NH conditions with less favorable and more stressful environmental conditions (higher CO_2_ and relative humidity levels) increase the physiological stress response to the exercise. However, the magnitude of the stress deviation is assimilable, at least in the ranges studied, and seems to benefit neural over structural adaptations for reasons that need to be studied. These results highlight the necessity of controlling environmental conditions and air quality when simulating NH conditions, mostly if the device used has limitations regarding the air flux. Therefore, the capacity of the hypoxia simulating device should be carefully considered when programming training periods.

## Data Availability

The datasets generated during and/or analyzed during the current study are available from the corresponding author on reasonable request.

## Abbreviations

R_T_: Resistance training
HH: Hypobaric hypoxia
FiO_2_: Inspired fraction of oxygen
NH: Normobaric hypoxia
PAO_2_: Alveolar pressure of oxygen
R: Respiratory exchange ratio
PACO_2_: Alveolar pressure of carbon dioxide
RSH: Repeated-sprint training in hypoxia
1RM: 1-repetition maximum
NHTent: Group that trained under NH conditions in a tent with poor air quality
NHRoom: Group that trained under NH conditions in a room with good air quality
HR: Heart rate
SpO_2_: Arterial oxygen saturation
SmO_2_: Muscle oxygen saturation
RPE: Rating of perceived exertion
SD: Standard deviation
ANOVA: Analysis of variance
η_p_^2^: Partial eta squared
ES: Cohen’s d effect size
